# Identification of Monotonically Differentially Expressed Genes for IFN-*β*-Treated Multiple Sclerosis Patients

**DOI:** 10.1155/2019/5647902

**Published:** 2019-12-12

**Authors:** Suyan Tian, Lei Zhang

**Affiliations:** ^1^Division of Clinical Research, The First Hospital of Jilin University, 71 Xinmin Street, Changchun, Jilin 130021, China; ^2^Department of Neurology, The Second Hospital of Jilin University, 218 Ziqiang Road, Changchun, Jilin 130041, China

## Abstract

Multiple sclerosis (MS) is a common neurological disability of the central nervous system. Immune-modulatory therapy with interferon-*β* (IFN-*β*) has been used as a first-line treatment to prevent relapses in MS patients. While the therapeutic mechanism of IFN-*β* has not been fully elucidated, the data of microarray experiments that collected longitudinal gene expression profiles to evaluate the long-term response of IFN-*β* treatment have been analyzed using statistical methods that were incapable of dealing with such data. In this study, the GeneRank method was applied to generate weighted gene expression values and the monotonically expressed genes (MEGs) for both IFN-*β* treatment responders and nonresponders were identified. The proposed procedure identified 13 MEGs for the responders and 2 MEGs for the nonresponders, most of which are biologically relevant to MS. Our work here provides some useful insight into the mechanism of IFN-*β* treatment for MS patients. A full understanding of the therapeutic mechanism will enable a more personalized treatment strategy possible.

## 1. Introduction

Multiple sclerosis (MS) is an immune-mediated, inflammatory demyelinating disease of the central nervous system with varying degrees of axonal loss, characterized by temporal and spatial dissemination of lesions [1]. MS can be categorized into relapsing-remitting and primary progressive. Approximately 85% of MS patients suffer from relapsing-remitting MS (RRMS) [2], where MS occurs repeatedly with a variety of symptoms, including multiple stages of neurological disability (relapse) and recovery (remission).

Immune-modulatory therapy with interferon-*β* (IFN-*β*) has been a commonly used first-line treatment to prevent relapses in RRMS patients [3]. For this, three-drug formulations of IFN-*β* are subcutaneous IFN-*β*-1a and IFN-*β*-1b and intramuscular IFN-*β*-1a. Nevertheless, the response rate of MS patients to IFN-*β* treatment is average, and within two years of the initial injection of IFN-*β*, nearly 40–50% of patients suffer from the first relapse [4], indicating poor or no response at all to the IFN-*β* treatments. While the therapeutic mechanism of IFN-*β* has not been fully elucidated, microarray experiments have been used to evaluate long-term response to IFN-*β* treatment. However, the resulting longitudinal gene expression profiles have usually been analyzed using statistical methods incapable of dealing with such data [5]. The inconsistency between the data and the analysis methods may cause biased or even totally incorrect conclusions, making it difficult to unravel the mechanism of action of IFN-*β* in MS. Therefore, a reanalysis of longitudinal gene expression data using a machine learning method capable of identifying genes that present a consistently changed pattern across time is recommended.

Feature selection is a machine learning method that downsizes the number of features, e.g., genes under consideration to a manageable level [6]. The gene subset identified by a feature selection usually has an optimal predictive capacity and relates highly to the phenotype of interest. Therefore, the identification of relevant gene expression profiles is usually accomplished with the aids of a feature selection method. Our previous studies [7–9] show that biological information contained within a pathway or a gene set (which presents a grouping structure so that the genes within the same gene set tend to coregulate or coexpress together to influence a biological process than those outside this gene set) may serve as a priori and provide valuable clues about the relevance of a gene. This holds true for both the cross-sectional feature selection processes [9] in which the data type is the gene expression profiles at a single time point and the longitudinal feature selection process [7] in which the gene expression profiles were collected subsequently across several time points. To decipher the therapeutic mechanism of IFN-*β*, a longitudinal feature selection that incorporates pathway information to guide the selection of genes presenting consistently changed patterns over time or, more precisely, the monotonically changing patterns over time are preferred.

In this study, the GeneRank method [10], an extension of Google's PageRank method [11], was used to analyze biomedical data and weigh the gene expression value as well as the level of importance of such a gene within the network to generate weighted gene expression values. Then, the monotonically expressed genes (MEGs) for both responders and nonresponders (of the IFN-*β* treatment) were identified, and their biological relevance was investigated. Those genes may provide some insightful clues about the therapeutic mechanism of IFN-*β* treatment and facilitate more personalized treatment strategies to MS patients.

## 2. Materials and Methods

### 2.1. Experimental Data

Data of two microarray experiments comprising one longitudinal data and one cross-sectional data were considered in this study. The statistical analyses carried out on these two datasets had different objectives. Using the cross-sectional data, the differentially expressed genes between MS patients and normal controls were identified. The objective of using the longitudinal data was to find MEGs for the responders and the nonresponders of IFN-*β* treatment.

Raw data (CEL file) of the MS temporal study were stored in the GEO (https://www.ncbi.nlm.nih.gov/geo/) repository (accession number GSE24427) [12]. For this experiment, longitudinal gene expression data were collected from 25 German relapsing-remitting multiple sclerosis patients treated with recombinant interferon-*β*-1b (rIFN-*β*-1b, 250 *μ*g administered every other day) for two years. The results of the analysis could potentially provide useful insights about molecular mechanisms of IFN-*β* intervention. In this study, we considered the corresponding data of the chips hybridized on the Affymetrix hgu133A platform.

Five separate time points were considered for measuring gene expression values, namely, before the first injection, before the second injection, before one month of injection, before 12 months of injection, and before 24 months of injection. The 25 patients were divided into two groups of 16 responders and 9 nonresponders, according to their first relapse time. Nevertheless, several patients relapsed shortly after the 2-year treatment duration and their gene expression profiles might have undergone some change during the treatment similar to what the nonresponders had experienced. Thus, to eliminate any ambiguity, we restricted the responder category to the patients whose first relapse time was more than five years (60 months) and finally listed nine responders (six females and three males) and nine nonresponders (six females and three males) for the downstream analysis. The first relapse time for these 25 patients, based on which the patients' response status to the IFN-*β* treatment was determined, is given in Table 1.

The cross-sectional MS data were acquired from the microarray data from the E-MTAB–69 experiment stored in the ArrayExpress [13] repository (http://www.ebi.ac.uk/arrayexpress). The chips of this experiment were hybridized on the Affymetrix HGU133 Plus 2.0 chips. In this study, 26 patients with RRMS and 18 controls with neurological disorders of noninflammatory nature were included.

### 2.2. Preprocessing Procedures

Raw data (CEL files) of the microarray analysis were downloaded from either the GEO repository (https://www.ncbi.nlm.nih.gov/geo/) or the ArrayExpress repository (http://www.ebi.ac.uk/arrayexpress). The expression values were obtained using the fRMA algorithm [14], normalized using quantile normalization, and then log 2 transformed.

### 2.3. Pathway Information

The information about gene-to-gene interaction/connection was retrieved from the Human Protein Reference Database (HPRD) [15], and the adjacency matrix was prepared based on these gene-to-gene interactions. There were 9,672 protein-coding genes annotated by the HPRD database, Release 9 (http://www.hprd.org). Among them, 7,473 genes were annotated by the R Bioconductor hgu133a.db package as well as the R Bioconductor hgu133plus2.db package.

## 3. Statistical Methods

### 3.1. GeneRank

The GeneRank method [10] calculates the ranks for genes by considering both the expression values and the connectivity information inside the gene-to-gene interaction network. Then, the GeneRank *r* solves the following equation:

(1) $$\begin{array}{c} \left(I_{p}-dW\;D^{-1}\right)r=\left(1-d\right){\text{exp}}_{i}, \end{array}$$

In equation (1), *I*_*p*_ is a *p *×* p* identity matrix. Here, *p* is the number of genes under consideration; *W* is an adjacency matrix which records how genes interplay with one another if the value of its *ij* component is 1 and then gene *i* and gene *j* are connected, and the value is 0 if gene *i* and gene *j* are not connected. *D* is also a *p *×* p* matrix, with its diagonal elements recording the degrees of freedom for these *p* genes and off-diagonal elements being zeroes. The degree of freedom is the number of genes to which the specific gene *k* (*k* = 1, 2,…, *p*) is connected, exp_*i*_ stands for gene expression values for sample *i* (*i* = 1, 2,…, *n*), and *d* is a tuning parameter, balancing off the influence of expression values and connectivity information on the ranking. The default value of 0.5 for *d* was used in this study.

The proposed procedure was conducted in three steps. First, the differentially expressed genes (DEGs) between MS patients and controls were identified by carrying out moderated *t*-tests with the help of the R limma package [16]. The issue of multiple testing was adjusted by using the Benjamini-Hochberg procedure [17], and the cutoff of adjusted *p* value was set at 0.1, a less stringent value compared to the default value of 0.05 because the sample size of the longitudinal microarray study was small. Then the GeneRanks of DEGs for each patient were calculated, which may be regarded as the weighted expression values of genes balancing between original expression values of the genes and their connectivity levels inside the gene-to-gene interaction network. Second, Kruskal-Wallis tests were carried out to compare if there were any differences in the expression value of one gene at different time points using the generated GeneRanks as outcome variables. The genes with adjusted *p* values less than a predetermined threshold (here, 0.1) were deemed as longitudinally differentially expressed genes across time points.

Among the longitudinal DEGs identified in step 2, the genes with specific patterns of change, i.e., monotonically expressed patterns, were screened out. A monotonically expressed gene was defined as

(2) $$\begin{array}{c} \bar{\;{\text{wexp}}_{1}}\;\leq \bar{{\text{wexp}}_{2}}\leq \bar{{\text{wexp}}_{3}}\leq \bar{{\text{wexp}}_{4}}\leq \bar{{\text{wexp}}_{5}},\\ \bar{{\text{wexp}}_{1}}\geq \bar{{\text{wexp}}_{2}}\geq \bar{{\text{wexp}}_{3}}\;\geq \bar{{\text{wexp}}_{4}}\geq \bar{{\text{wexp}}_{5}\;}. \end{array}$$

If the weighted expression values (i.e., the generated GeneRanks) of a gene at different time points satisfy the strictly “not less than” condition, then a gene presents the monotonically increasing expressed (MIE) change pattern. In contrast, if the weighted expression levels of a gene follow the strictly “not more than” trend, then a gene was deemed as a monotonically decreasing expressed (MDE) gene. Here, $\bar{{\text{wexp}}_{k}}\;$ represents the average weighted expression value of a specific gene, i.e., the average of generated GeneRanks at time point *k*, where *k* = 1, 2,…, 5.

**Figure 1:**
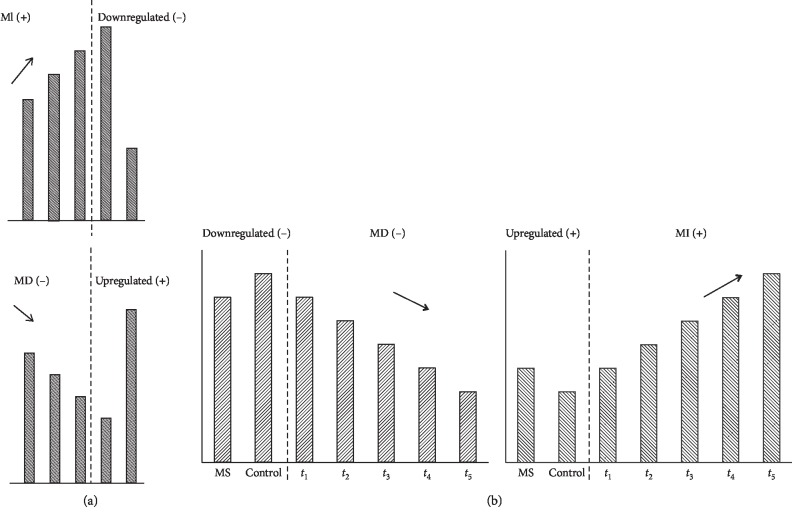
Graphical illustration on a “good response” gene and a “bad response” gene. (a) The definition of a “good response” gene. (b) The definition of a “bad response” gene. From these plots, it can be seen that the expression differences decreased over a period of time for a “good response” gene while such differences increase further over a period of time for a “bad response” gene.

For responders and nonresponders separately, one set of MIE genes and one set of MDE genes were identified. Then, if an underexpressed DEG for MS versus control was an MIE gene as well or an overexpressed DEG (MS versus control) was an MDE gene as well, this gene was regarded as a “good response” gene. These “good response” genes may provide mechanistic insights into IFN-b treatment. Contrarily, a “bad response” gene corresponded to an overexpressed MIE gene or an underexpressed MDE gene. Having more such genes could suggest the ineffectiveness of the treatment and that the MS gradually worsens to relapse. The concepts of “response” and “no response” genes are graphically illustrated in Figure 1.

## 4. Results

### 4.1. DEGs and MEGs

When the significance level (the cutoff for the adjusted *p* value) was set at 0.1, 1078 genes were identified to be downregulated and 288 genes were deemed as upregulated in MS patients (as per the cross-sectional MS data). Interestingly, most DEGs were downregulated (nearly 3 times the number of overexpressed genes).

In these DEGs (identified using the cross-sectional MS data), the GeneRanks were calculated, and then steps 2 and 3 of the proposed procedure were applied to identify MEGs for the nonresponder and the responder groups, respectively. The identified MEGs are listed in Table 2, in which the directions of both differential expression change (MS versus control) and monotonic expression change pattern (over time) are presented as well. There were 13 MEGs for the responders and two MEGs for the nonresponders, respectively. As per our definitions of “good response” and “bad response” genes in the previous section, the responders possessed eight “good response” gene and five “bad response” genes, and both MEGs identified in the nonresponder group were “bad response” genes. In MS, all “good response” genes possessed by the responders and all “bad response” genes possessed by the nonresponders were underexpressed. Among the “bad response” genes possessed by the responders, all except *IGLL1* were overexpressed in MS.

### 4.2. Biological Relevance

According to the GeneCards database [18], among these eight “good response” genes, four genes (i.e., *MYD88*, *LILRB1*, *ALOX5*, and *AFTPH*) were associated directly and the remaining genes were associated indirectly with MS. Among the four directly associated genes, two of them, i.e., *MYD88* and *LILRB1,* were reported to correlate with the IFN-*β* treatment. Specifically, Myeloid Differentiation Primary Response 88 (*MYD88*) encodes a cytosolic adapter protein that plays a central role in the innate and adaptive immune responses. This protein functions as an essential signal transducer in the interleukin-1 and Toll-like receptor signaling pathways, which regulate the activation of several proinflammatory genes. A study by Zhang et al. [19] demonstrated that IFN-*β*-1a treatment is able to increase the expression of *MYD88*, mediated by IFN-*β*-1a-induced *TLR7* expression in dendritic cells (DCs). This is consistent with the results of our analysis, which revealed a monotonic increase in *MYD88* expression during the first two years of IFN-*β* injection among the responders.

Leukocyte Immunoglobulin-Like Receptor B1 (*LILRB1*) is a member of the Leukocyte Immunoglobulin-Like Receptor (LIR) family. It controls inflammatory responses and cytotoxicity to help focus on the immune response and limit autoreactivity. A study [20] pointed the association of LILRB1 with MS/IFN-*β* treatment and the expression of different NKR by NK cells and CD8+ T lymphocytes from MS patients and healthy controls were analyzed by flow cytometry. The MS patients with active clinical disease displayed lower levels of LILRB1+ NK cells compared to those in controls or nonactive MS patients, and progressive MS patients displayed higher levels of LILRB1+ CD8+ T lymphocytes and LILRB1+ NK cells compared to those in RRMS patients. Their analysis further showed a negative association between IFN-*β* therapy and LILRB1 expression by CD8+ T lymphocytes and NK cells. Their results seem to have several contradictions; i.e., if LILRB1 expression in MS active patients was lower than that in healthy people and inactive patients, it was natural to expect the IFN-*β* therapy to be positively related to LILRB1 expression (to eliminate the difference between MS patients and normal controls). Furthermore, compared to RRMS patients, the LILRB1 expression was found to increase in the progressive MS patients. However, IFN-*β* therapy is the first-line treatment for RRMS, so the subpopulation for these two comparisons may differ. In addition, there was no variation between responders and nonresponders to the treatment. It is highly likely that the changes in expression of this gene in these two groups were in contrast to each other. A long-term epidemiologic study to investigate the role of LILRB1 in IFN-*β* therapy for MS is warranted.

Among the “bad response” genes in the responders, two genes, namely, phosphatase and tensin homolog (*PTEN*) and CHM rab escort protein (*CHM*), were found directly related to MS according to the GeneCards database. Several previous studies have verified the association of *PTEN* with MS. For instance, Meira et al. [21] showed the downregulation of miR-17 with the treatment and its upregulation during relapse by comparing miR-17 expressions in CD4+ T cells from relapsing-remitting (RR) MS patients treated with natalizumab versus the patients without this treatment. They also found that the miR-17 downregulation was associated with the upregulation of *PTEN*, *BIM*, *E2F1*, and *p21* target genes. However, for *CHM,* no such studies have been reported. Of note, at the least stringent cutoff of 0.2 for adjusted *p* values, Janus Kinase 2 (*JAK2*) may be regarded as a “bad response” gene among the responders as well, although it is not so at a cutoff of 0.1. It is directly related to MS and IFN-*β*. According to the GeneCards database, JAK2 is involved in various processes such as cell growth, development, differentiation, or histone modifications. It mediates essential signaling events in both innate immunity and adaptive immunity. In the cytoplasm, JAK2 plays a pivotal role in signal transduction via its association with type I receptors such as growth hormone (GHR) or with type II receptors including IFN-*α*, IFN-*β*, IFN-*γ*, and multiple interleukins [22]. To effectively inhibit the expansion of Th17 cells by IFN-*β*, intact IFN-*γ* signaling in T cells is required. In Conti's study [23], the authors reported that both mRNA and cell surface expression of the signaling chain of the IFN-*γ* receptor (IFN-*γ*R2) and its cognate tyrosine kinase JAK2 were enhanced in peripheral blood Th17 cells and clones from MS patients, compared to those with inactive multiple sclerosis or healthy controls.

Among the “bad response” genes in the nonresponders, only MMS19 Homolog, Cytosolic Iron-Sulfur Assembly Component (*MMS19*) was found directly related to MS but had no association with the IFN-*β* treatment according to the GeneCards database [18]. Therefore, we conjecture that those “bad response” genes may at most serve as an indicator of worsening disease and cannot provide more information on the mechanism of the IFN-*β* treatment or response. Further investigation is needed to justify this conjecture.

### 4.3. Enriched Pathways and Interaction Network

**Figure 2:**
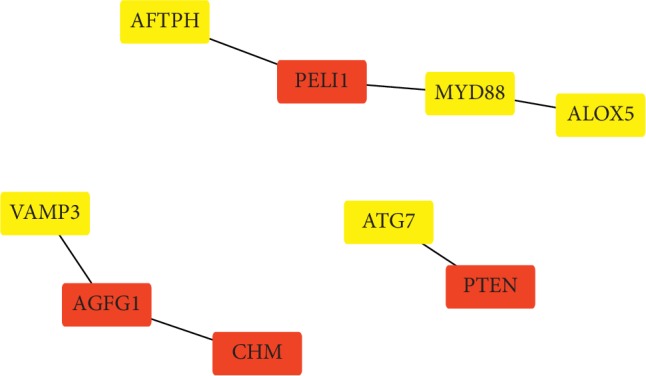
Gene-to-gene interaction network for the MEGs identified by the proposed procedure. In this diagram, the interaction information was retrieved using the String software, and on the basis of the gene-to-gene interaction information, the Cytoscape software was used to plot the network. On this network, the isolated genes were omitted, and “good response” genes were highlighted in yellow and “bad response” genes were highlighted in red.

For the identified MEGs, enriched pathways or gene ontology (GO) terms [24] were obtained using the String software [25]. There were 92 GO biological process (BP) terms and 17 Reactome [26] pathways enriched by these MEGs. Notably, the most significant five GO BP terms include immune response, defense response, cellular response to an organic substance, positive regulation to immune response, and protein transport, most of which are related to an autoimmune response. Focusing on the “good response” genes, the overrepresented analysis in the String software identified 41 enriched GO BP terms, eight GO cellular component (CC) terms, one KEGG [27] pathway, and nine Reactome [26] pathways. Moreover, most of these enriched pathways/gene sets are related to immune responses. In Figure 2, the gene-to-gene interaction network was constructed using the Cytoscape software [28] based on the interaction information downloaded from the String web page (https://string-db.org).

## 5. Conclusions

This study identified “good response” and “bad response” genes to the IFN-*β* treatment for RRMS patients, with the aid of bioinformatics tools to obtain the DEGs and MEGs. One big limitation of this study was the small sample size (with nine responders and nine nonresponders). A large-sized longitudinal gene expression study is highly recommended.

To conclude, this study provides useful insights into the therapeutic mechanism of IFN-*β* treatment that selectively targets the autoimmune response in MS patients. Only with a full understanding of such a therapeutic mechanism, a more personalized treatment strategy is possible.

## Figures and Tables

**Table 1:** First relapse time for the patients in longitudinal multiple sclerosis study.

Category	Patient ID number
First relapse >60 months	2, 3, 5, 9, 14, 16, 19, 24, 25
First relapse 24∼60^*∗*^	8, 10, 20, 1, 4, 13, 18
First relapse <24 months	6, 7, 11, 12, 15, 17, 21, 22, 23

^*∗*^Those patients were excluded from the data analysis.

**Table 2:** MEGs identified by the proposed method.

Responders						Nonresponders		
“Good response” genes			“Bad response” genes			“Bad response” genes		
Symbol	MEG	DEG	Symbol	MEG	DEG	Symbol	MEG	DEG
AFTPH	↑	↓	AGFG1	↑	↑	NAP1L4	↓	↓
ALOX5	↑	↓	CHM	↑	↑	MMS19	↓	↓
ATG7	↑	↓	IGLL1	↓	↓			
MYD88	↑	↓	PELI1	↑	↑			
LILRB1	↑	↓	PTEN	↑	↑			
PRKAB1	↑	↓						
PSEN1	↑	↓						
VAMP3	↑	↓						

MEGs ↑: monotonically increasing genes over time; MEGs ↓: monotonically decreasing genes over time; DEGs ↑: overexpressed genes (MS versus control); DEGs ↓: underexpressed genes (MS versus control). For the discordant genes, the directions of monotonic expression change and differential expression change are opposite to each other; over a period of time, the gene expression values tend to return to the expression levels of normal controls. For the concordant genes, the directions of monotonic expression change and differential expression change are identical to each other; over a period of time, the gene expression values tend to deviate more away from the expression levels of normal controls.

## Data Availability

The longitudinal experimental data (accession number: GSE24427) were downloaded from the Gene Expression Omnibus (GEO) repository (https://www.ncbi.nlm.nih.gov/geo/) and the cross-sectional experimental data (experimental number: E-MTAB-69) were downloaded from the ArrayExpress repository (http://www.ebi.ac.uk/arrayexpress).
